# Assessing the performance of a novel Finnish register-based measure of precarious employment: affected employee groups and subjective and objective employment outcomes

**DOI:** 10.1186/s12889-026-26520-3

**Published:** 2026-02-04

**Authors:** Taina Leinonen, Laura Salonen, Theo Bodin, Svetlana Solovieva

**Affiliations:** 1 Finnish Institute of Occupational Health, Helsinki, Finland; 2 Institute of Environmental Medicine, Karolinska Institutet, Stockholm, Sweden; 3 Center for Occupational and Environmental Medicine, Region Stockholm, Stockholm, Sweden

**Keywords:** Precarious employment, Register data, Measurement, Sociodemographic factors, Occupational group, Job insecurity, Unemployment

## Abstract

**Background:**

As an important driver of inequality in the labor market, it is crucial to develop measures to assess precarious employment. Most previous measures have been survey-based.

**Methods:**

We used register data on wage-earners aged 20–64 residing in Finland in 2013 (*N* = 1 873 210) to develop a novel measure of precarious employment, including items on job discontinuity, multijob holding, agency employment, underemployment, and employment income. We assessed the performance of the measure by examining the distribution of precarious employment by sociodemographic factors and occupation as well as exploring its associations with subjective (job insecurity with survey information from the same year 2013 linked to the register data at an occupational-group-level) and objective (unemployment over a 5-year follow-up) employment outcomes using linear regression.

**Results:**

In the total study population, approximately 5% had precarious employment. It was more prevalent among women, younger age groups, individuals with a foreign background, those with lower education, manual workers, and private sector employees. Large-size occupations with more than 10% of precariously employed individuals included teachers’ aides, waiters, food service counter attendants, car, taxi and van drivers, security guards, kitchen helpers, and cooks. Precarious employment was not consistently associated with subjective job insecurity. It was nevertheless associated with objective occurrence and duration of unemployment.

**Conclusions:**

Precarious employment, as captured by the novel Finnish register-based measure, has similar background factors as recognized in previous literature and identifies occupations expected to be characterized by precarious employment. Those classified as precariously employed based on objective characteristics of their employment relationships do not necessarily perceive their job as insecure, although they have a clearly increased risk of subsequent unemployment. The measure can be considered to perform well for use in further studies applying Finnish register data.

**Supplementary Information:**

The online version contains supplementary material available at 10.1186/s12889-026-26520-3.

## Background

Previous literature has defined precarious employment in various ways, but generally, it refers to aspects of nonstandard employment, which may have negative consequences for individuals’ health and wellbeing [1–7]. In this paper, we approach precarious employment as a multidimensional concept that reflects circumstances where several unfavourable features of employment relationships accumulate for the individual. In a systematic review [5] of definitions and operationalizations of precarious employment, such features were categorized under three main dimensions: (1) employment insecurity, which may include e.g. temporary, agency, and part-time employment as well as multiple job holding; (2) income inadequacy; and (3) lack of rights and protection, which may cover e.g. lack of collective bargaining power, regulatory protection, workplace rights, and social security. According to our multidimensional approach, non-standard employment such as temporary or part-time employment is considered as precarious only when it is accompanied by other unfavourable features of employment relationships, for example low income level.

Precarious employment is unevenly distributed across the working population. It is found to be more common, for example, among younger employees, women, those in lower socioeconomic positions and those with immigrant background [8–16]. Precarious employment is also associated with other unfavourable labour market outcomes; however, approaches have varied regarding whether adverse working conditions, subjective experiences of job insecurity, and unemployment have been addressed as a part of the precarious employment measure or as linked independent factors [8, 10, 11, 16–22]. Not being intrinsic to employment relationships themselves [4], we conceptualize subjective job insecurity and unemployment not as inherent aspects of precarious employment, but as distinct outcomes that can be influenced by it.

As an important form of inequality in the labor market, it is crucial to develop measures to assess precarious employment in studies. Most previous measures of precarious employment have been based on survey data, including, for example, the validated Employment Precariousness Scale [8] which has been adapted to several countries [23–25] and to cross-European data [26]. It is also important to develop measures of precarious employment using register data, which offer objective information on several aspects of employment relationships. Such data provide opportunities to assess predictors and outcomes of precarious employment based on large nationally representative study populations and longitudinal designs. In Nordic countries, register data have been applied to one or more single aspects of precarious employment such as job instability, multiple job holding, part-time work and earnings [27–30]. Register-based multidimensional measures of precarious employment were first developed in Sweden [12]. Two approaches were applied: one based on typologies and another based on a summative score. The latter approach served as the basis for developing a corresponding measure for Finland in the current paper, including items on job discontinuity, multijob holding, agency employment, underemployment, and employment income. Similar items were included in the Swedish version, except for underemployment. Moreover, the Swedish version included an item on unionization, for which information was lacking in Finland.

A common way of assessing the performance of precarious employment measures has been to examine their association with known background factors, such as sociodemographic and occupational factors [8–10, 14, 17]. Furthermore, the performance of a precarious employment measure can be assessed in terms of predictive validity, i.e. by examining its association with expected outcome measures. Such outcomes have included, for example, subjective job insecurity, psychosocial work characteristics, and health and wellbeing [8, 16].

The aims of this paper were to (1) introduce a multidimensional measure of precarious employment using Finnish register data, and assess the performance of the measure by (2) examining its distribution across sociodemographic groups and occupations, and (3) exploring its associations with subjective (job insecurity in one’s occupational group with information from a previously conducted survey linked to the register data) and objective (unemployment over a 5-year follow-up) employment outcomes.

## Data and methods

### Study population

We utilized register-based FOLK data from Statistics Finland, which includes the total Finnish population with pseudonymized information on sociodemographic factors, occupation, employment and industrial sector, employer-specific employment episodes, unemployment days, as well as sources of income from work, entrepreneurship, and different types of social security benefits.

Since the survey including information on subjective job insecurity was conducted in 2013, the register-based measure of precarious employment was also applied to year 2013 in this paper. The measure can nevertheless be applied from year 2008 onwards until the most recent year for which register information has been updated.

For this study, we included employed wage earners aged 20–64 who were residing in Finland at the end of the observation year 2013. The requirement for inclusion was that the individuals had at least one employment day and income from wage employment (rounded to the nearest 100 euros) during the observation year (initially 2 409 216 individuals). Despite being employed wage earners, we excluded the following groups (defined in Supplementary Table 1): (1) students (10.3%), (2) primarily self-employed (4.9%), (3) pensioners (3.6%), (4) those with major leaves from work due to sickness (0.4%) or child care (2.0%), and (5) recent migrants (0.8%). Although students, self-employed, and pensioners may engage in precarious employment, the current general measure of precariousness is not suitable for these specific groups. Individuals on sickness or child care leaves may have an ongoing employment relationship but not receive employment income, which would lead to underestimation of the level of income from employment when assessing precariousness. Recent migrants lack information on the three-year history of employment relationships required to assess precariousness. After these exclusions, the study population consisted of 1 873 210 individuals.

### Precarious and other employment types

Before assessing employment precariousness, we identified individuals with limited employment, defined as those who had more than 180 days of unemployment (4.9%) or less than 90 days of employment (0.9%) during the observation year, or did not have at least one employer both during the observation year and the two preceding years (3.7%). For those with limited employment, it is challenging to differentiate the contribution of precarious employment from risk factors associated with being outside employment. Moreover, information on employers over three consecutive years was used for the precarious employment measure (see below). Limited employment may share elements with precarious employment and was included in the data as a separate category of employment type.

For the remaining 1 696 370 individuals, employment type was determined based on a precarious employment score. As the basis for calculating this score, we used the previously developed Swedish measure applying the multidimensional summative score approach [12], more specifically its second version [e.g. 13]. However, we made certain modifications to accommodate the content of the available data in Finnish registers. Differences compared to the Swedish version are presented in Supplementary Table 2. Of the three main dimensions of the applied precarious employment concept [5], our measure captures aspects of employment insecurity (job discontinuity, multijob holding, agency employment, and underemployment items) and income inadequacy (employment income item), but not lack of rights and protection, which is more difficult to assess with register data.

The precarious employment score was calculated as the sum of values assigned to the five different items. Distributions and assigned values of the items are presented in Table 1 and described below. Negative values indicate elements of precariousness.

- *Job discontinuity* was defined as having three different employers or at least two new main employment episodes during the observation and the two preceding years, based on the longest employment episode of each year (“No” = 0, “Yes” = −2). The main employment episode was considered new if there was at least one day between that and the previous one.
- *Multijob holding* was defined as having three or more employers or further three or more employers in three or more main industrial sectors during the observation year, either simultaneously or successively (“No” = 0, “Different employers” = −1, “Different employers in different sectors” = −2).
- *Agency employment* was defined as having employment in the industry “temporary employment agency activities” during the observation year but not having the occupation “employment agents and contractors” (“No” = 0, “Yes” = −1). The information was based on the employment episode of the end of the year, and if not available, the longest episode of the year (only available for the industrial sector) or that of the end of the previous year.
- *Underemployment* was based on employment income per employed day in the observation year, relative to the median income among the population included in the calculation of the precarious employment score, within strata by occupation, age group, gender, and employment sector (“No underemployment” = 0, “≥1/3 and < 2/3 of median” = −1, “<1/3 of median” = −2). Such income-based magnitude of work contribution in relation to one’s peers aimed to capture part-time or irregular employment, which was used as a proxy for underemployment. The age strata used were 20–34, 35–49, and 50–64. Information on occupation and employment sector was derived from the end of the observation year, and if unavailable, it was based on the most recent information from the three previous years. The employment sector was divided into private and public, and in cases where the sector was unknown, it was defined as private. For occupation, we used a classification by Statistics Finland from year 2010, based on the International Standard Classification of Occupations. The occupational codes included up to five digits and could be determined for most individuals included in the calculation of the precarious employment score (99.3%). For remaining individuals, we used educational level (primary, secondary, tertiary) instead of occupation to form the strata. For strata with less than 30 individuals (1.2%), we combined the employment sectors, genders and age groups, and finally, used occupational codes at a less specific digit level until the stratum size reached at least 30.
- *Employment income* was based on employment income per employed day in the observation year, relative to the median income among the whole population included in the calculation of the precarious employment score (“≥200% of median” = 2, “≥120%, < 200% of median” = 1, “≥80%, < 120% of median” = 0, “≥60%, < 80% of median” = −1, “<60% of median” = −2).

**Table 1:** Distribution and assigned score values of the precarious employment items, excluding those with limited employment

Precarious employment item	*N*	%	Score value
Job discontinuity			
No	1 510 945	89.1	0
Yes	185 425	10.9	−2
Multijob holding			
No	1 608 959	94.9	0
Jobs	52 058	3.1	−1
Jobs and sectors	35 353	2.1	−2
Agency employment			
No	1 674 327	98.7	0
Yes	22 043	1.3	−1
Underemployment			
No	1 538 984	90.7	0
≥ 1/3 and < 2/3 of median	127 969	7.5	−1
< 1/3 of median	29 417	1.7	−2
Employment income			
≥ 200% of median	115 386	6.8	2
≥ 120%, < 200% of median	433 803	25.6	1
≥ 80%, < 120% of median	702 560	41.4	0
≥ 60%, < 80% of median	286 359	16.9	−1
< 60% of median	158 262	9.3	−2
Total	1 696 370	100.0	

The correlation between underemployment and employment income was moderate (*r* = 0.465), while correlations between other item combinations were weak (ranging between 0.068 and 0.219) (Supplementary Table 3). Based on the precarious employment score, calculated as the sum of the values of the five items, we formed the following employment type categories: High income (> 0), Standard (0), Sub-standard (−3–−1), and Precarious (<−3). To be defined as precariously employed, an individual thus had to receive the value − 2 from two different items (e.g. job discontinuity and employment income below 60% of the median) or values − 1 and − 2 from a combination of items summing up to −4 or below (e.g. multiple jobs (in multiple sectors), at least some level of underemployment, and employment income at least below 80% of the median). Higher employment income was considered as a protective factor, and the positive values assigned to it could partly cancel out the negative values assigned to elements of precariousness (e.g. job discontinuity and multiple jobs in multiple sectors in combination with employment income 120% or above the median would be defined as sub-standard instead of precarious employment). Similar principals for these categorizations have been used in the Swedish version of the measure [20, 22, 31].

### Sociodemographic factors

As background factors, we examined gender, age (5-year age groups, some combined for descriptive purposes), origin (Finnish background, foreign background) and attained education (tertiary, secondary, primary) in the observation year 2013. We also examined the most recent occupational class (upper non-manual, lower non-manual, skilled manual, unskilled manual, other) and employment sector (private, public) measured at the end of 2013 or the five preceding years as well as unemployment history based on the total number of unemployment days during the years 2009–2013 (no unemployment, 1–90 days, 91–365 days, > 365 days).

### Subjective and objective employment outcomes

Subjective job insecurity and the occurrence and duration of unemployment were examined as potential outcomes of precarious employment.

#### Subjective job insecurity

Occupation-level information on subjective job insecurity was derived from the Quality of Work Life Survey (QWLS) from 2013. The QWLS is an extensive personal interview survey conducted by Statistics Finland at five-year intervals since the 1970 s to monitor employees’ working conditions and changes in them. The monthly Finnish Labour Force Survey (FLFS), which is a random selection from the population register applied by region in proportion to population weights, serves as source population for the QWLS. Wage and salary earners aged 15–64 years who normally work at least 10 h per week are selected from the FLFS population and invited to participate in the QWLS. For the QWLS 2013, around 7000 wage and salary earners were selected from the second, third, fourth and fifth rotation groups of the FLFS in September and October 2013. Accounting for non-participation in both the FLFS and QWLS, the response rate was 69%. The data were collected through personal face-to-face interviews using a standardized questionnaire.

In the QWLS 2013, job insecurity was assessed with the question: “Are the following factors of uncertainty related to your work?”. Responding “Yes” to at least one of the response alternatives “Threat of dismissal” or “Threat of unemployment” was considered as perceiving one’s job as insecure. We calculated the proportion of employees experiencing subjective job insecurity by occupational group, based on the same occupational classification of Statistics Finland from 2010 as in the register data, but available only at a 3-digit level. Out of 130 3-digit occupational groups, 42 (32%) had fewer than 10 respondents. For these groups, the proportion of employees experiencing job insecurity was imputed using aggregated values from 2-digit-level occupational groups. For example, the occupational group with code 131 (Production managers in agriculture, forestry and fisheries) had fewer than 10 respondents, while occupational groups coded 132 (Manufacturing, mining, construction, and distribution managers), 133 (Information and communications technology service managers), 134 (Professional services managers) each had more than 10 respondents. Imputation was made for 131 code, the imputed value being equal to the proportion of employees experiencing job insecurity within the occupational group with code 13 (Production and specialised services managers, combining respondents from 131, 132, 133, and 134).

The occupational-group-level information on the proportion of employees experiencing job insecurity derived from the QWLS 2013 was then linked to individuals in the register data based on their occupational codes. Individuals in the register-based study population for whom occupation could be determined at a 3-digit level (*N* = 1 826 211, 97.5%, being 99.3% among those who were included in the calculation of the precarious employment score and 80.5% among those with limited employment) were included in the analyses on job insecurity.

#### Occurrence and duration of unemployment

Information on unemployment was derived from the register data and followed up during the years 2014–2018, subsequent to the measurement of precarious employment in 2013. We considered unemployment occurrence based on having more than 30 days of unemployment as well as the total number of unemployment days during the 5-year follow-up. The 30-day threshold was applied because unemployment lasting around one month or less may indicate a transition between jobs rather than a lack of employment. The analyses on unemployment applied to the same study population as those on subjective job insecurity, i.e. individuals with a defined occupation. Additionally, individuals were required to reside in Finland throughout the 5-year follow up period (*N* = 1 798 882, 98.5% of those with a defined occupation). Furthermore, when examining the number of unemployment days, only individuals with any unemployment during the follow-up were examined (*N* = 439 592, 24.4% of those who were followed up for unemployment occurrence).

### Statistical analyses

For descriptive purposes, we plotted a heatmap indicating quartiles of the proportions of employees with precarious employment, subjective job insecurity, and unemployment occurrence by 3-digit occupational group.

Linear regression analysis was used to examine the association of precarious employment and other employment types with subjective job insecurity and the occurrence and duration of unemployment. Based on marginal means of the models, we calculated the predicted proportion of employees with subjective job insecurity, unemployment occurrence and the predicted number of unemployment days during the 5-year follow-up by employment type. The models were adjusted for age group, gender, origin, and education. In the unemployment analyses, unemployment history was additionally adjusted for, and in a supplementary analysis, it was also examined as an effect modifier.

## Results

### Distribution of precarious employment

Within the study population, 4.9% were classified as precariously employed (Table 2). The proportion with limited employment, for whom the precarious employment score could not be calculated, was even larger at 9.4%. Both precarious and limited employment were more prevalent among younger age groups, individuals with a foreign background, those with lower education, manual workers, private sector employees, and those with a history of unemployment. The share of precariously employed was slightly higher among women, whereas limited employment was more common among men.

**Table 2:** Percentage distribution of employment type by sociodemographic factors

	Employment type					
	High income	Standard	Sub-standard	Precarious	Limited	Total
All	26.9	33.1	25.8	4.9	9.4	100.0
Gender						100.0
Men	35.2	30.2	19.7	4.6	10.3	100.0
Women	18.3	36.0	31.9	5.3	8.6	100.0
Age						
20–24	1.4	12.5	40.2	18.6	27.3	100.0
25–34	16.7	33.1	33.4	6.2	10.6	100.0
35–44	32.1	32.4	23.9	3.9	7.7	100.0
45–54	33.1	35.7	21.1	3.1	7.0	100.0
55–64	30.8	36.8	21.5	2.8	8.0	100.0
Origin						
Finnish background	27.5	33.5	25.3	4.7	8.9	100.0
Foreign background	13.6	22.9	34.0	9.0	20.5	100.0
Education						
Tertiary	48.8	25.3	17.0	3.3	5.5	100.0
Secondary	18.9	37.1	28.6	5.3	10.1	100.0
Primary	13.1	31.5	33.0	6.9	15.5	100.0
Occupational class						
Upper non-manual	60.1	19.1	12.7	3.4	4.8	100.0
Lower non-manual	19.4	40.9	28.1	4.4	7.2	100.0
Skilled manual	15.6	38.0	28.8	5.8	11.8	100.0
Unskilled manual	4.5	23.1	46.9	8.6	16.9	100.0
Other	1.7	2.7	14.2	10.3	71.1	100.0
Employment sector						
Private	26.8	30.1	26.2	5.9	11.0	100.0
Public	27.0	38.5	24.9	3.1	6.5	100.0
5-year unemployment history						
No unemployment	34.9	38.5	21.9	2.9	1.7	100.0
1–90 days	17.0	33.3	36.7	7.0	6.1	100.0
91–365 days	9.6	22.7	38.5	10.1	19.2	100.0
> 365 days	2.4	7.6	25.5	10.4	54.2	100.0
N	503 017	619 016	482 329	92 008	176 840	1 873 210

Among the precariously employed, nearly all had employment income below 80% of the median, and over 80% had it below 60% of the median (Supplementary Table 4). Approximately 70% experienced some level of underemployment. Two-thirds had job discontinuity, one-third held multiple jobs, and over 10% were employed through an agency. The majority (63.5%; 69.1% among men and 58.5% among women) scored negative values for three or more of the five items, multiple factors thereby contributing to being defined as precariously employed (Supplementary Table 5). When only two items contributed negative values, employment income was typically below 60% of the median in combination with underemployment below one-third of the median (19.9%; 20.1% among men and 19.7% among women) or with job discontinuity (12.5%; 6.4% among men and 18.0% among women). Other combinations with only two contributing items were rare.

Various cultural occupations appeared at the top of the list in terms of having highest proportions of precarious employees (Table 3). However, these were relatively small occupations. Among occupations where more than 10% were precariously employed, the largest ones were teachers’ aides and several service and sales occupations, including waiters, food service counter attendants, car, taxi and van drivers, security guards, kitchen helpers, and cooks.

**Table 3.**
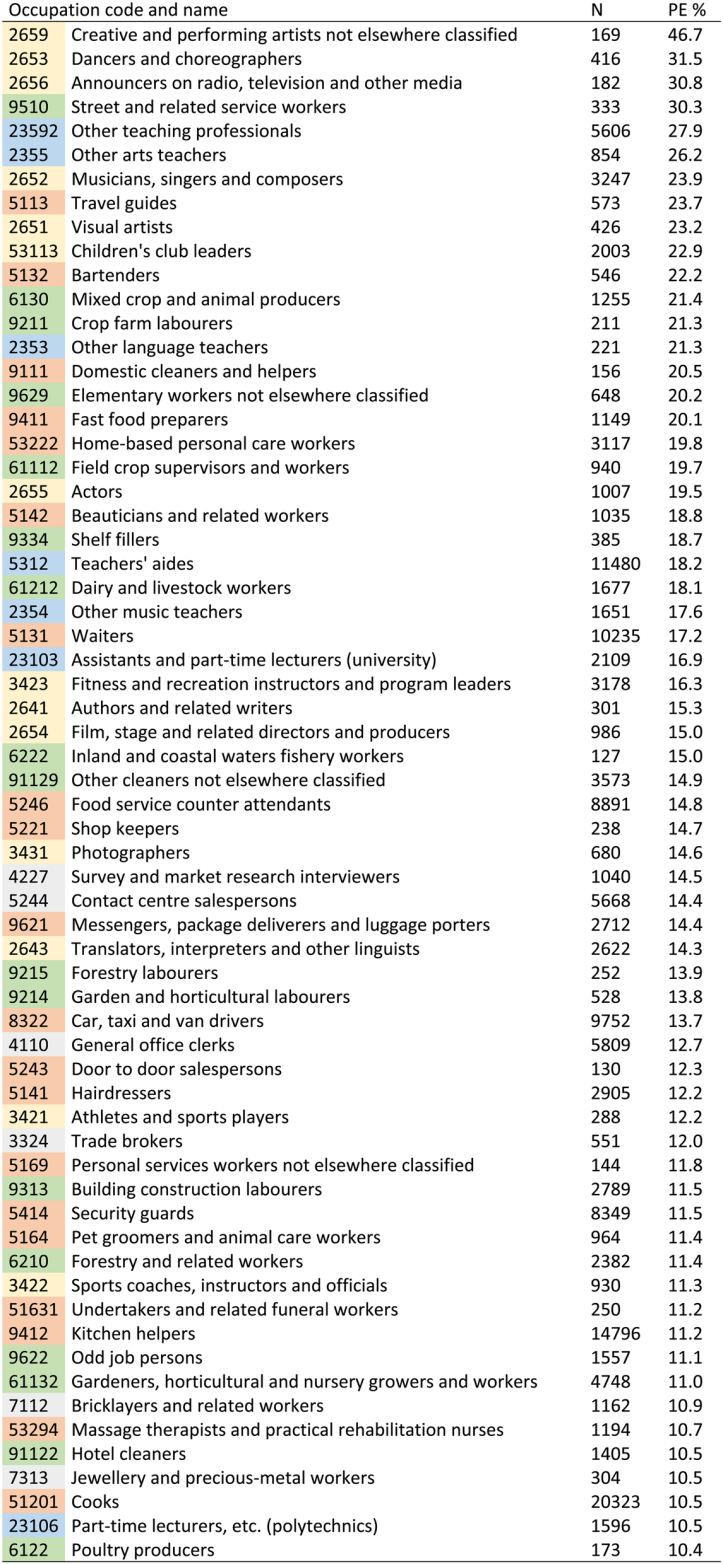
Occupations in which more than 10% of employees had precarious employment (PE). The colours represent categorization into “cultural and sports-related” (yellow), “diverse teaching” (blue), “services and sales” (red), “primary production and elementary” (green), and other (grey) occupations

Occupations with less than 100 individuals excluded

**Fig. 1:**
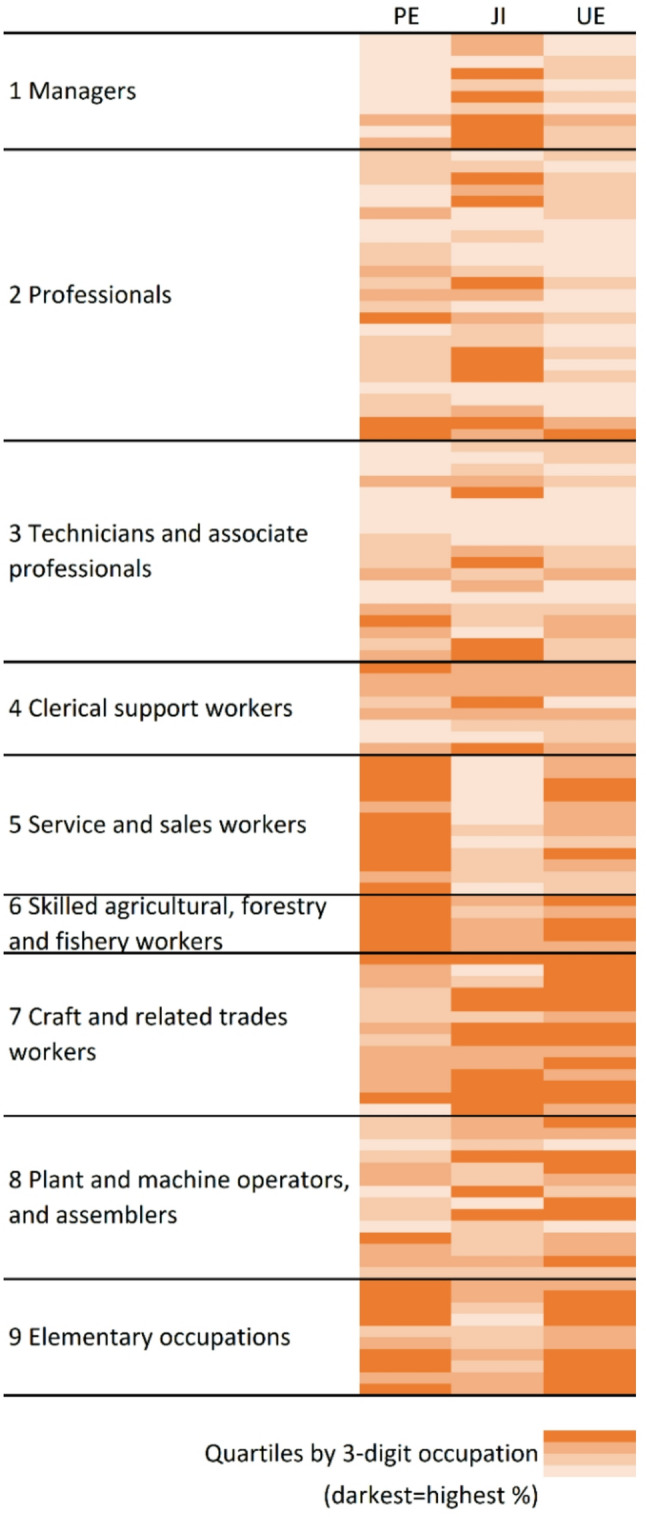
Precarious employment (PE), subjective job insecurity (JI) and unemployment occurrence (UE) by occupational group. Distribution of 3-digit occupational groups into quartiles in terms of the percentage of employees with PE, JI and UE with each 3-digit group representing one of the thin lines, excluding military occupations and occupational groups in which the percentage is based on less than 3 individuals

The 3-digit occupational groups that belonged to the quartile with highest proportions of precarious employees were most commonly found in major job categories 5 Service and sales workers, 6 Skilled agricultural, forestry and fishery workers, and 9 Elementary occupations (Fig. 1). More detailed information on these 3-digit occupational groups is presented in Supplementary Table 6. Many top-quartile occupational groups in terms of the proportion of employees with unemployment occurrence were in these same major job categories, but also in 7 Craft and related trades workers and 8 Plant and Machine operators, and assemblers. For the proportion of employees with subjective job insecurity, the top-quartile occupational groups were more widespread across different levels of occupational categories, appearing also/more in the major job categories 1 Managers, 2 Professionals, and 3 Technicians and associate professionals. Top-quartile occupational groups in terms of subjective job insecurity were most prevalent in 7 Craft and related trades workers, where also unemployment was common, but did not appear in 5 Service and sales workers, 6 Skilled agricultural, forestry and fishery workers, and 9 Elementary occupations, which were the major job categories where precarious employment was most prevalent.

It is important to note that there was also variation in precarious employment within the occupational groups. Within the 3-digit occupational groups in the top quartile, some specific occupations had relatively low proportions of precarious employees (around 3% or less), such as education methods specialists and special needs teachers (within Other teaching professionals), shop supervisors (within Shop salespersons), police officers and prison guards (within Protective services workers), as well as travel attendants and travel stewards and transport conductors (within Travel attendants, conductors and guides), each of these occupations including at least 500 individuals (Supplementary Table 7).

### Precarious employment and further employment outcomes

Based on the information by occupational group, 24.4% of employees were defined to have subjective job insecurity. The proportion of unemployment occurrence during the five-year follow-up was also 24.4%, the mean number of unemployment days being 479.

Based on the regression models, precarious employment was not clearly associated with the predicted proportion of subjective job insecurity, which was lowest for standard employment and highest for high-income employment, with sub-standard, precarious, and limited employment falling in between (Table 4). The differences between employment types were small, all within a range of 2% points.

**Table 4:** Predicted proportion of employees with subjective job insecurity, unemployment and predicted unemployment days by employment type

Employment type	Predicted % subjective job insecurity, *N* = 1 826 211 (a)	Predicted % unemployment occurrence during 5 years, *N* = 1 797 882 (a, b)	Predicted unemployment days during 5 years, *N* = 439 592 (a, b, c)
High income	25.8	19.1	420
Standard	23.8	20.2	407
Sub-standard	24.1	26.8	444
Precarious	24.4	37.5	496
Limited	24.9	46.3	631

Adjusted for age, gender, origin and education, and in the unemployment analyses also for unemployment history

(a) Including individuals with a defined 3-digit occupation

(b) Including individuals who remain in the country during the whole 5-year follow-up

(c) Including individuals with unemployment occurrence during the 5-year follow-up

The predicted proportion of the five-year unemployment occurrence was largest for limited employment at 47.3%, followed by precarious employment at 38.0%, and the lowest for high-income employment at 18.6%. The differences in the predicted number of unemployment days followed the same pattern, with 631, 496, and 420 days over the five years, respectively. The higher occurrence and duration of unemployment for precarious and limited employment remained when stratifying by categories of unemployment history (Supplementary Table 8).

## Discussion

We introduced a register-based measure of precarious employment by adapting the previously developed Swedish measure [12] to Finnish data. Our new measure can be considered to perform well in terms of its prevalence among sociodemographic groups previously recognized as more commonly exposed to precarious employment and in identifying occupations expected to be characterized by precarious employment. Although our precarious employment measure was not consistently associated with subjective job insecurity, this is likely not due to poor performance of the measure but because job insecurity reflects a more widespread uncertainty in the labour market, as elaborated below. The precarious employment measure performed well in predicting objective occurrence and duration of unemployment.

### Prevalence of precarious employment and differences between employee groups

In the total study population, approximately 5% had precarious employment in the observation year 2013. Using the corresponding measure, the proportion in Sweden in 2012 was found to be around 13–14% depending on gender [13]. This difference may partly result from including those with less than three months of employment in a separate category of limited employment in Finnish study population. It may also relate to differences in how the measure was constructed. The Swedish version of the job discontinuity item was based on having at least one change of main employer over three years. The Finnish data additionally included information on the onset of new main employment episodes, which can also be with the same employer. Information on both changes in the employer and onsets of new employment episodes were utilized in the Finnish version of job discontinuity, requiring at least two points of discontinuity in one of these. The item was thus defined more strictly in the Finnish than in the Swedish version. We considered that only one change in employer or a new employment episode might capture also normal career advancement. In Finland in 2013, around 15% of wage earners had temporary employment [32]. The around 11% experiencing job discontinuity in our data may be in line with this, since we could not identify temporary employment episodes immediately following previous ones with the same employer. Using a similar definition of job discontinuity as in Sweden and excluding those with limited employment from the denominator would result in around 7% of the Finnish study population being classified as having precarious employment. The Finnish version also includes an item on underemployment instead of lack of unionization, thereby measuring a different aspect of precarious employment compared to the Swedish version. Besides measurement differences, there may be actual differences in the prevalence of precarious employment between the countries. Previous comparative studies on European countries nevertheless indicate that precarious employment is among the least common in both Finland and Sweden [10, 18, 26].

A previous study using Finnish survey data reported a precarious employment proportion of 13% in 2013 [11]. The prevalences of single items on temporary and/or agency employment as well as low income were in line with what we found. However, the previous study included also prevalent items on the threat of layoff, dismissal and/or unemployment, on the assessment of chances of finding a new job in the open labour mark, and on realized unemployment, which we consider outcomes of precarious employment rather than inherent aspects of it.

In line with previous studies [8–16], we found that the share of precariously employed was largest among younger age groups, those with a foreign background, those with lower education, manual workers, and private sector employees. Although precarious employment was somewhat higher among women than among men, the opposite was found for limited employment, which may share elements with precarious employment and thus dilute the gender difference. Moreover, among the precariously employed, a larger number of unfavourable features contributed among men. One factor that may have partly reduced the disadvantages faced by women is that a notable share of women was employed in the public sector, which had less precarious and limited employment than the private sector. The traditionally found gender difference may also be shifting. Previous findings from Sweden indicate that between 2012 and 2017 men surpassed women in the share of precariously employed [13].

Our measure also identified occupations which we expect to be characterized by precarious employment, such as certain creative and teaching occupations as well as service and sales occupations. We found that large occupations with large shares of precariously employed individuals included teachers’ aides, waiters, food service counter attendants, car, taxi and van drivers, security guards, kitchen helpers, and cooks. Similar top-ranking occupations have been indicated previously [12, 15]. Other studies based on broader categories of occupation or industrial sectors have shown precarious employment to be common in: service occupations [14] or sector [10]; the sector of accommodation and food services and that including professional, scientific and technical activities as well as administrative and support service activities [12]; the sectors of primary production, construction, manufacturing, education and research, as well as health care and social work [11]; and the sectors of trade and transport as well as culture, leisure, and other services [16]. Comparison of these findings is difficult due to considerable differences both in the precarious employment measures and in the categorization of the employment fields.

### Further employment outcomes

Based on our findings, subjective job insecurity was slightly more common in precarious than standard employment, but most common in high-income employment. Subjective job insecurity was relatively widespread across different occupational levels, being prevalent also in many high-skilled occupational groups. However, those commonly exposed to precarious employment, such as service and sales occupations, did not have the highest shares of job insecurity. In occupations where non-standard employment is common, precarious employment deviates less from the norm, and thus may not result in subjective feelings of insecurity as much as would be expected based on objective employment characteristics. Previous studies from other countries have nevertheless found positive, although not necessarily strong, associations between precarious employment and subjective job insecurity [8, 16]. Our findings indicate that at least in the Finnish context, subjective job insecurity relates to much broader labour market circumstances than precarious employment. These may include, for example, recurring restructuring and layoffs in organizations, affecting a wide range of occupations. In such circumstances, individuals in presumably advantageous positions may feel their achieved high status in the labour market threatened, even though their actual risk of job loss or unemployment would be relatively small. According to Finnish results based on the QWLS, subjective job insecurity has largely fluctuated with economic cycles [32]. Nevertheless, since the turn of the millennium both upper non-manual employees and manual workers have generally reported higher shares experiencing threats of dismissal and unemployment — i.e. components of job insecurity used in the current paper — compared to lower non-manual employees. Prior literature not only from Finland [32] but also more broadly [33, 34] suggests that while previously job insecurity was predominantly linked to less-skilled manual work, in the past decades it has become more prevalent among non-manual employees, affecting also those in high-skilled managerial and professional positions.

We found that precarious employees had a clearly increased risk of unemployment occurrence and a larger number of unemployment days during a five-year follow-up. The association between precarious employment and unemployment has not been extensively investigated. When addressed in previous studies, unemployment has typically been considered part of the precarious employment measure [11, 17, 18] or as a separate exposure category [19]. However, in accordance with our findings, a study from Sweden found that precariously employed young adults had an increased risk of long-term unemployment during middle-adulthood [20]. While we approach precarious employment as adverse circumstances related to employment relationships rather than non-employment, unemployment may have contributed to people ending up in precarious employment. Furthermore, previous experiences of unemployment may have influenced subsequent unemployment outcomes of precarious employees. Pre-existing experiences of unemployment were, nevertheless, unlikely to fully explain our findings on the association between precarious employment and unemployment during follow up, since the results also applied to individuals without any unemployment in the measurement year of precarious employment or the four preceding years.

### Strengths and limitations

The strengths of the newly introduced precarious employment measure include its application to nationally representative register data with objective information on features of employment relationships, avoiding selection bias. It involves a large study population, enabling the examination of specific occupations, and provides a reliable follow-up for unemployment with no loss to follow-up. Furthermore, we complemented the register data with survey information on occupational-group-specific job insecurity, allowing us to assess a subjective employment outcome along with the objective one based on unemployment.

Moreover, the precarious employment measure is based on an existing Swedish measure, which is theoretically grounded and has been used in numerous studies examining trends in precarious employment [13] and its associations with health [21, 22, 31] and labour market [20] outcomes. The item on underemployment was not included in the original Swedish measure due to lack of data. However, underemployment, often approached through part-time employment, is theoretically driven [2, 6, 7] and commonly incorporated in previous measures of precarious employment [5]. Our underemployment item is similar to the measure of partial work contribution previously developed for Finnish register data [35]. It was shown to capture a realistic magnitude of work participation among people receiving full or partial disability pension, who are thereby only allowed to earn only up to a specified threshold.

A low correlation between most of the items in our precarious employment measure does not imply poor performance; rather, it reflects the heterogeneous nature of precarious employment, where different aspects may contribute. Thus, the different elements of precarious employment may not always co-occur [8, 11, 16]. In our measure, low income and underemployment contributed among the majority of precarious employees, yet most were exposed to three or more unfavourable features of employment relationships.

One shortcoming of our precarious employment measure is that it does not include items on the lack of rights and protection. In Finland, most employees are covered by collective agreements [36, 37], so lack of bargaining power may contribute less to precarious employment than in some other countries. However, our measure should be interpreted as reflecting aspects of employment insecurity and low income, thus not necessarily being comparable to other precarious employment measures, especially those in which the lack of rights and protection play a large role. Furthermore, our measure was built on objective aspects of employment relationships and is thus likely to reflect different circumstances than measures based on subjective experiences of precariousness [38, 39].

The use of register data also has other limitations. For instance, although the item on job discontinuity covered gaps between employment periods and changes in the employer, we could not capture successive temporary employment contracts with the same employer. This may have partly contributed to the inconsistent association found between precarious employment and subjective job insecurity. We may also have partly misclassified underemployment, since the income-based magnitude of work contribution in relation to one’s peers may not always reflect the actual amount of work done. Moreover, we do not know whether the individuals defined as having job discontinuity, multiple jobs, agency employment, or underemployment did so voluntarily, which would not necessarily indicate precariousness. Finally, the five items of precarious employment were measured at different time frames (job discontinuity during the observation years and two preceding years, multijob holding, employment income, and underemployment during the observation year, and agency employment at the end of the observation year) depending on availability of the data, which is why the individuals did not necessarily experience these conditions simultaneously.

The measure of subjective job insecurity was based on occupational-level information from the previously conducted QWLS. Individuals with disadvantageous positions in the labour market may have been less likely to participate in the survey. Furthermore, the aggregated measure did not capture variation in job insecurity between individuals within an occupation. These issues may have undermined the examined association between precarious employment and job insecurity. However, our analyses indicated that there was little overlap between the occupational groups with a high share of precariously employed and those with a high share experiencing subjective job insecurity. A strong individual-level association between precarious employment and job insecurity may therefore not necessarily be expected.

### Future research directions using the measure

The precarious employment measure introduced in this paper provides a novel opportunity to assess precarious employment in studies using Finnish register data. Our work adds to the register-based approaches to precarious employment, which provide possibilities to examine the phenomenon using large nationally representative data and longitudinal designs. The applied summative score approach defines a single category for precariousness, which may be useful when examining, for example, time trends, distribution in the population, differences between countries, or when complex study designs are applied. The summative score approach would also be suitable for developing a job exposure matrix (JEM), i.e. information on the prevalence of precarious employment in strata by gender, age group, sector, and occupation. The JEM could be linked to corresponding employee strata in large-scale studies that do not have the opportunity to measure precarious employment at the individual level. In the future, typological approaches could additionally be used for the Finnish register data to capture more homogeneous groups of people experiencing specific aspects of precariousness.

Our precarious employment measure is applicable to the general population of wage earners in Finland but not to all employee groups. For example, investigations of working students [40] and the self-employed [12, 14, 41] require other types of conceptualizations and operationalizations of precarious employment. Moreover, a sufficient amount of employment is required to be able to assess aspects of employment relationships using the measure. A relatively large proportion, over 9% of the study population, had employment which was too limited to ascertain whether it was precarious. We did not, for example, capture precariousness of seasonal workers employed less than 90 days during the year. In future studies, limited employment can be examined as a separate category or excluded, depending on the study aim and design.

In future research, the precarious employment measure can be applied to other study years than the current 2013. Moreover, the register data allows for examining longer-term circumstances such as trajectories or cumulative experiences of precarious employment.

## Conclusions

Precarious employment, as captured by the novel Finnish register-based measure, has similar background factors as recognized in previous literature and identifies occupations expected to be characterized by precarious employment. Those classified as precariously employed based on objective characteristics of their employment relationship do not necessarily perceive their job as insecure. They do, nevertheless, have a clearly increased risk of subsequent unemployment, even without previous experience of unemployment. The precarious employment measure can be considered to perform well for use in further studies applying Finnish register data.

## Supplementary Information

Supplementary Material 1.

## Data Availability

The register data used in this study are not publicly available. Researchers can apply permission from Statistics Finland to access the data (https:/stat.fi/tup/tutkijapalvelut/kayttoluvan-hakeminen-ja-lupamuutokset_en.html).

## Abbreviations

QWLS: Quality of Work Life Survey
FLFS: Finnish Labour Force Survey
JEM: Job exposure matrix
