# Supplementary material for: Assessing the performance of a novel Finnish register-based measure of precarious employment: affected employee groups and subjective and objective employment outcomes
Source: BMC Public Health. 2026 Feb 4;26:807. doi: 10.1186/s12889-026-26520-3 (PMC12964599; doi:10.1186/s12889-026-26520-3)
Supplement: Supplementary file 1 — Supplementary Material 1. [file 12889_2026_26520_MOESM1_ESM.pdf]

**Supplementary Table 1.** Groups excluded from the study population in the observation year 2013

| Excluded group | Description | % |
|---|---|---|
| Students | Main activity at the end of the observation year student or receiving student allowance during the observation year | 10.3 |
| Primarily self-employed | Socioeconomic status at the end of the observation year self-employed or entrepreneurial income in the observation year higher than work income | 4.9 |
| Pensioners | Main activity at the end of the observation year pensioner or receiving any type of pension other than survivor's pension during the observation year | 3.6 |
| Those with major leaves from work | Sum of sickness allowance and parental and other child home care allowances being more than 50% of one's employment income in the observation year | 0.4 and 2.0 due to sickness and child care, respectively, based on whichever allowance was the highest |
| Recent migrants | Did not reside in the country at the end of the observation year or two preceding years | 0.8 |

**Supplementary Table 2.** Operationalization of precarious employment items applied to wage earners using register data in the current Finnish version and the previously developed Swedish version of the measure

| Finnish version | | Swedish version | | |
|---|---|---|---|---|
| Item name | Description | Item name | Description | Notes |
| Job discontinuity | Having at least three different employers or at least two new main employment episodes during the observation and the two preceding years, based on the longest employment episode of each year | Contractual temporariness | Having at least two different employers during the observation and the two preceding years, based on the employer from which largest income was gained in each year | Finnish version stricter, leading to a smaller and more selected group for this item |
| Multijob holding | Having three or more employers or further three or more employers in thee or more main industrial sectors during the observation year | Multiple jobs/ economic sectors | Having three or more employers or further three or more employers in thee or more main industrial sectors during the observation year | Similarly measured |
| Agency employment | Having the longest employment of the observation year in the industry "temporary employment agency activities", but not having the occupation "employment agents and contractors" | Contractual relationship insecurity | Having the employer from which largest income was gained during the observation year in the industry "temporary employment agency activities" | No large discrepancy expected |
| Under-employment | Employment income per employed day in the observation year in relation to the median income of the study population in strata by occupation, age group, gender and employment sector | | | Not included in the Swedish version, which may lead to differences between the countries in what the precarious employment measure captures |

| | | | | |
|---|---|---|---|---|
| Employment income | Employment income per employed day in the observation year in relation to the median income of the whole study population | Income level | The sum of employment income and work-related benefits elevated to the level the income would be if salary were paid | Although differently measured, both versions examine the income level from employment while being actively working, so no large discrepancy expected |
| | | Lack of unionization | Proportion of employees being covered by collective bargaining agreements in the inductrial sector of the employer from which largest income was gained | Not included in the Finnish version, which may lead to differences between the countries in what the precarious employment measure captures |

**Supplementary Table 3.** Correlations between the precarious employment items

| | Job discontinuity | Multijob holding | Agency employment | Underemployment | Employment income |
|---|---|---|---|---|---|
| Job discontinuity | 1.000 | | | | |
| Multijob holding | 0.152 | 1.000 | | | |
| Agency employment | 0.112 | 0.071 | 1.000 | | |
| Underemployment | 0.125 | 0.105 | 0.068 | 1.000 | |
| Employment income | 0.219 | 0.086 | 0.089 | 0.465 | 1.000 |

**Supplementary Table 4.** Distribution and assigned score values of the precarious employment items among those with precarious employment

| Precarious employment item | N | % | Score value |
|---|---|---|---|
| Job discontinuity | | | |
| No | 30 779 | 33.4 | 0 |
| Yes | 61 229 | 66.6 | -2 |
| Multijob holding | | | |
| No | 61 194 | 66.5 | 0 |
| Jobs | 13 863 | 15.1 | -1 |
| Jobs and sectors | 16 951 | 18.4 | -2 |
| Agency employment | | | |
| No | 82 245 | 89.4 | 0 |
| Yes | 9 763 | 10.6 | -1 |
| Underemployment | | | |
| No | 27 701 | 30.1 | 0 |
| ≥1/3 and <2/3 of median | 35 285 | 38.4 | -1 |
| <1/3 of median | 29 022 | 31.5 | -2 |
| Employment income | | | |
| ≥200% of median | 0 | 0.0 | 2 |
| ≥120%, <200 % of median | 36 | 0.0 | 1 |
| ≥80%, <120% of median | 3 247 | 3.5 | 0 |
| ≥60%, <80% of median | 14 116 | 15.3 | -1 |
| <60% of median | 74 609 | 81.1 | -2 |
| Total | 92 008 | 100.0 | |

**Supplementary Table 5.** Percentage distribution of combinations of the five precarious employment items (job discontinuity, multijob holding, agency employment, underemployment, and employment income) contributing among men and women defined as precariously employed

| Contributing precarious employment items | Men | Women | All |
|---|---|---|---|
| Two items only | | | |
| Job discontinuity & multiple jobs in multiple sectors | 3.7 | 1.9 | 2.8 |
| Job discontinuity & employment income below 60% of the median | 6.4 | 18.0 | 12.5 |
| Multiple jobs in multiple sectors & employment income below 60% of the median | 0.7 | 1.9 | 1.3 |
| Underemployment below one-third of the median & employment income below 60% of the median | 20.1 | 19.7 | 19.9 |
| Other combinations of two items | <0.1 | <0.1 | <0.1 |
| Three items only | | | |
| Job discontinuity, multiple jobs (in multiple sectors) & employment income at least below 80% of the median | 7.2 | 9.9 | 8.6 |
| Job discontinuity, agency employment & employment income at least below 80% of the median | 3.3 | 2.6 | 2.9 |
| Job discontinuity, at least some underemployment & employment income at least below 80% of the median | 31.5 | 23.0 | 27.0 |
| Multiple jobs (in multiple sectors), at least some underemployment & employment income at least below 80% of the median | 9.2 | 9.6 | 9.4 |
| Agency employment, at least some underemployment & employment income at least below 80% of the median | 2.1 | 1.6 | 1.8 |
| Other combinations of three items | 1.3 | 1.0 | 1.2 |
| Four or five items | 14.5 | 10.8 | 12.5 |
| Total, % | 100.0 | 100.0 | 100.0 |
| N | 43 308 | 48 700 | 92 008 |

**Supplementary Table 6.** Percentage of employees (darker shades represent quartiles with higher %) with precarious employment (PE), job insecurity (JI) and unemployment occurrence (UE) by 3-digit occupational group, excluding military occupations and occupational groups in which the percentage is based on less than 3 individuals

| Occupational group code and name | | N | PE % | JI % | UE % |
|---|---|---|---|---|---|
| 111 | Legislators and senior officials | 3684 | 1.3 | 25.9 (I) | 8.7 |
| 112 | Managing directors and chief executives | 2055 | 0.7 | 25.9 (I) | 13.7 |
| 121 | Business services and administration managers | 9027 | 1.1 | 10.5 | 16.8 |
| 122 | Sales, marketing and development managers | 12445 | 0.8 | 32.1 | 20.5 |
| 132 | Manufacturing, mining, construction, and distribution managers | 11828 | 1.1 | 22.6 | 16.6 |
| 133 | Information and communications technology service managers | 4638 | 0.7 | 66.7 | 17.3 |
| 134 | Professional services managers | 14073 | 1.2 | 23.4 | 8.4 |
| 141 | Hotel and restaurant managers | 597 | 3.7 | 33.3 (I) | 24.2 |
| 142 | Retail and wholesale trade managers | 3484 | 1.2 | 33.3 (I) | 17.9 |
| 143 | Other services managers | 945 | 4.8 | 33.3 (I) | 19.5 |
| 211 | Physical and earth science professionals | 3660 | 2.7 | 11.1 | 20.3 |
| 212 | Mathematicians, actuaries and statisticians | 824 | 3.4 | 27.1 (I) | 13.6 |
| 213 | Life science professionals | 7924 | 3.3 | 31.0 | 19.6 |
| 214 | Engineering professionals (excluding electrotechnology) | 41059 | 1.4 | 27.8 | 17.8 |
| 215 | Electrotechnology engineers | 18232 | 1.6 | 36.2 | 21.9 |
| 216 | Architects, planners, surveyors and designers | 10004 | 4.4 | 13.0 | 21.2 |
| 221 | Medical doctors | 14593 | 1.3 | 8.3 | 1.9 |
| 222 | Nursing and midwifery professionals | 5348 | 0.8 | 23.1 | 3.2 |
| 225 | Veterinarians | 688 | 2.8 | 12.2 (I) | 6.6 |
| 226 | Other health professionals | 6361 | 2.4 | 13.8 | 7.4 |
| 231 | University and higher education teachers | 14435 | 5.0 | 24.5 | 13.3 |
| 232 | Vocational education teachers | 14596 | 3.4 | 34.0 | 18.9 |
| 233 | Secondary education teachers | 21014 | 4.1 | 25.0 | 15.7 |
| 234 | Primary school and early childhood teachers | 39248 | 3.5 | 12.7 | 14.9 |
| 235 | Other teaching professionals | 23569 | 10.9 | 26.5 | 24.0 |
| 241 | Finance professionals | 16701 | 1.6 | 18.2 | 12.8 |
| 242 | Administration professionals | 24608 | 2.8 | 23.2 | 15.6 |
| 243 | Sales, marketing and public relations professionals | 27785 | 2.7 | 37.0 | 22.3 |
| 251 | Software and applications developers and analysts | 29635 | 2.0 | 32.1 | 16.7 |
| 252 | Database and network professionals | 5338 | 2.4 | 33.3 (I) | 19.5 |
| 261 | Legal professionals | 6941 | 1.8 | 15.8 | 5.8 |
| 262 | Librarians, archivists and curators | 3299 | 3.1 | 10.0 | 13.1 |
| 263 | Social and religious professionals | 19654 | 3.3 | 25.4 | 13.6 |
| 264 | Authors, journalists and linguists | 10826 | 7.1 | 38.1 | 24.6 |
| 265 | Creative and performing artists | 6603 | 22.8 | 28.6 | 36.0 |
| 311 | Physical and engineering science technicians | 52882 | 1.6 | 24.4 | 18.1 |
| 312 | Mining, manufacturing and construction supervisors | 8456 | 1.4 | 16.0 | 22.8 |
| 313 | Process control technicians | 2637 | 1.5 | 24.2 (I) | 14.4 |
| 314 | Life science technicians and related associate professionals | 5434 | 4.3 | 29.4 | 23.6 |
| 315 | Ship and aircraft controllers and technicians | 2811 | 1.9 | 40.0 | 16.3 |
| 321 | Medical and pharmaceutical technicians | 12151 | 1.8 | 2.3 | 7.3 |
| 322 | Nursing and midwifery associate professionals | 56119 | 1.7 | 15.9 | 6.6 |
| 324 | Veterinary technicians and assistants | 383 | 1.0 | 13.5 (I) | 10.9 |

| | | | | | |
|---|---|---|---|---|---|
| 325 | Other health associate professionals | 13296 | 3.5 | 16.3 | 11.3 |
| 331 | Financial and mathematical associate professionals | 24882 | 2.2 | 28.4 | 17.1 |
| 332 | Sales and purchasing agents and brokers | 47623 | 3.5 | 37.5 | 22.9 |
| 333 | Business services agents | 13841 | 5.5 | 23.5 | 24.7 |
| 334 | Administrative and specialised secretaries | 24723 | 1.8 | 25.8 | 15.7 |
| 335 | Regulatory government associate professionals | 17901 | 1.4 | 9.6 | 5.9 |
| 341 | Legal, social and religious associate professionals | 34574 | 4.6 | 19.4 | 20.5 |
| 342 | Sports and fitness workers | 4488 | 15.0 | 20.0 (I) | 30.0 |
| 343 | Artistic, cultural and culinary associate professionals | 5437 | 6.4 | 15.4 | 25.5 |
| 351 | Information and communications technology operations and user support technicians | 19684 | 2.2 | 43.1 | 20.5 |
| 352 | Telecommunications and broadcasting technicians | 4807 | 4.1 | 44.6 (I) | 21.8 |
| 411 | General office clerks | 5809 | 12.7 | 28.6 | 29.4 |
| 412 | Secretaries (general) | 37320 | 4.6 | 29.0 | 26.0 |
| 413 | Keyboard operators | 1516 | 4.6 | 29.8 (I) | 25.5 |
| 421 | Tellers, money collectors and related clerks | 16074 | 2.2 | 34.0 | 16.6 |
| 422 | Client information workers | 16983 | 6.2 | 29.8 | 26.8 |
| 431 | Numerical clerks | 16966 | 2.0 | 20.0 | 18.4 |
| 432 | Material-recording and transport clerks | 10834 | 1.9 | 17.4 | 22.6 |
| 441 | Other clerical support workers | 25510 | 5.2 | 32.7 | 24.9 |
| 511 | Travel attendants, conductors and guides | 2721 | 7.2 | 16.6 (I) | 31.5 |
| 512 | Cooks | 32654 | 7.9 | 16.1 | 28.5 |
| 513 | Waiters and bartenders | 10971 | 17.4 | 11.1 | 40.2 |
| 514 | Hairdressers, beauticians and related workers | 3940 | 14.0 | 16.6 (I) | 35.3 |
| 515 | Building and housekeeping supervisors | 25885 | 5.0 | 18.0 | 24.7 |
| 516 | Other personal services workers | 2205 | 8.7 | 16.6 (I) | 25.5 |
| 522 | Shop salespersons | 94788 | 7.6 | 20.5 | 27.2 |
| 523 | Cashiers and ticket clerks | 7676 | 9.9 | 14.3 | 23.9 |
| 524 | Other sales workers | 19541 | 12.9 | 23.5 | 36.0 |
| 531 | Child care workers and teachers' aides | 48839 | 8.4 | 18.7 | 32.7 |
| 532 | Personal care workers in health services | 93469 | 4.2 | 20.9 | 18.5 |
| 541 | Protective services workers | 18881 | 7.3 | 14.3 | 17.2 |
| 611 | Market gardeners and crop growers | 5961 | 12.5 | 26.7 | 40.9 |
| 612 | Animal producers | 7079 | 10.0 | 24.0 | 27.2 |
| 613 | Mixed crop and animal producers | 1255 | 21.4 | 27.3 (I) | 35.7 |
| 621 | Forestry and related workers | 2382 | 11.4 | 27.5 (I) | 61.3 |
| 622 | Fishery workers, hunters and trappers | 447 | 10.3 | 27.5 (I) | 33.0 |
| 711 | Building frame and related trades workers | 45736 | 7.9 | 36.4 | 54.8 |
| 712 | Building finishers and related trades workers | 21567 | 4.6 | 17.1 | 43.6 |
| 713 | Painters, building structure cleaners and related trades workers | 6855 | 6.2 | 18.2 | 53.3 |
| 721 | Sheet and structural metal workers, moulders and welders, and related workers | 17880 | 3.5 | 37.8 | 49.0 |
| 722 | Blacksmiths, toolmakers and related trades workers | 23055 | 2.5 | 33.3 | 34.2 |
| 723 | Machinery mechanics and repairers | 38273 | 2.8 | 22.8 | 25.8 |
| 731 | Handicraft workers | 1689 | 5.3 | 45.5 (I) | 34.7 |
| 732 | Printing trades workers | 6036 | 2.9 | 50.0 | 41.3 |
| 741 | Electrical equipment installers and repairers | 23426 | 4.5 | 25.8 | 31.2 |
| 742 | Electronics and telecommunications installers and repairers | 9454 | 4.5 | 26.3 | 33.2 |
| 751 | Food processing and related trades workers | 4134 | 5.4 | 31.6 (I) | 24.6 |
| 752 | Wood treaters, cabinet-makers and related trades workers | 8601 | 5.2 | 36.8 | 46.8 |

| | | | | | |
|---|---|---|---|---|---|
| 753 | Garment and related trades workers | 2759 | 6.7 | 31.6 (I) | 45.3 |
| 754 | Other craft and related workers | 2431 | 1.4 | 31.6 (I) | 27.6 |
| 811 | Mining and mineral processing plant operators | 7675 | 2.7 | 28.6 | 40.2 |
| 812 | Metal processing and finishing plant operators | 6255 | 2.1 | 30.9 (I) | 25.5 |
| 813 | Chemical and photographic products plant and machine operators | 7885 | 2.0 | 20.0 | 16.6 |
| 814 | Rubber, plastic and paper products machine operators | 8601 | 2.2 | 33.3 | 33.5 |
| 815 | Textile, fur and leather products machine operators | 5996 | 5.9 | 18.2 | 39.1 |
| 816 | Food and related products machine operators | 13583 | 4.8 | 24.1 | 26.2 |
| 817 | Wood processing and papermaking plant operators | 17164 | 1.6 | 42.4 | 24.0 |
| 818 | Other stationary plant and machine operators | 9373 | 3.3 | 17.6 | 33.6 |
| 821 | Assemblers | 20494 | 3.2 | 32.0 | 35.7 |
| 831 | Locomotive engine drivers and related workers | 3229 | 0.7 | 21.9 (I) | 7.4 |
| 832 | Car, van and motorcycle drivers | 9851 | 13.7 | 23.5 | 30.3 |
| 833 | Heavy truck and bus drivers | 43461 | 4.3 | 20.0 | 28.3 |
| 834 | Mobile plant operators | 20215 | 5.8 | 27.9 | 38.6 |
| 835 | Ships' deck crews and related workers | 1432 | 3.0 | 21.9 (I) | 23.6 |
| 911 | Domestic, hotel and office cleaners and helpers | 63385 | 9.1 | 27.7 | 32.1 |
| 912 | Vehicle, window, laundry and other hand cleaning workers | 292 | 7.5 | 27.7 (I) | 34.3 |
| 921 | Agricultural, forestry and fishery labourers | 1085 | 14.8 | 24.8 (I) | 50.3 |
| 931 | Mining and construction labourers | 9382 | 7.2 | 5.3 | 47.7 |
| 932 | Manufacturing labourers | 3254 | 2.8 | 18.9 (I) | 31.7 |
| 933 | Transport and storage labourers | 32784 | 6.3 | 20.6 | 31.4 |
| 941 | Food preparation assistants | 15945 | 11.8 | 29.3 | 37.3 |
| 951 | Street and related service workers | 333 | 30.3 | 24.8 (I) | 42.4 |
| 961 | Refuse workers | 1283 | 5.1 | 26.7 (I) | 57.8 |
| 962 | Other elementary workers | 4953 | 14.0 | 28.6 | 45.9 |

(I) = Imputed value

**Supplementary Table 7.** Proportion of employees with precarious employment in specific occupations within the top quartile of 3-digit occupational groups, excluding occupations with less than 100 individuals

| Occupational code and name | | % | N |
|---|---|---|---|
| 951 | Street and related service workers | 30.3 | 333 |
| – | No further digits applied | | |
| | | | |
| 265 | Creative and performing artists | 22.8 | 6 603 |
| 2651 | Visual artists | 23.2 | 426 |
| 2652 | Musicians, singers and composers | 23.9 | 3 247 |
| 2653 | Dancers and choreographers | 31.5 | 416 |
| 2654 | Film, stage and related directors and producers | 15.0 | 986 |
| 2655 | Actors | 19.5 | 1 007 |
| 2656 | Announcers on radio, television and other media | 30.8 | 182 |
| 2659 | Creative and performing artists not elsewhere classified | 46.8 | 169 |
| | | | |
| 613 | Mixed crop and animal producers | 21.4 | 1 255 |
| – | No further digits applied | | |
| | | | |
| 513 | Waiters and bartenders | 17.4 | 10 971 |
| 5131 | Waiters | 17.2 | 10 235 |
| 5132 | Bartenders | 22.2 | 546 |
| | | | |
| 342 | Sports and fitness workers | 15.0 | 4 488 |
| 3421 | Athletes and sports players | 12.2 | 288 |
| 3422 | Sports coaches, instructors and officials | 11.3 | 930 |
| 3423 | Fitness and recreation instructors and program leaders | 16.3 | 3 178 |
| | | | |
| 921 | Agricultural, forestry and fishery labourers | 14.8 | 1 085 |
| 9211 | Crop farm labourers | 21.3 | 211 |
| 9214 | Garden and horticultural labourers | 13.8 | 528 |
| 9215 | Forestry labourers | 13.9 | 252 |
| | | | |
| 962 | Other elementary workers | 14.0 | 4 953 |
| 9621 | Messengers, package deliverers and luggage porters | 14.4 | 2 712 |
| 9622 | Odd job persons | 11.1 | 1 557 |
| 9629 | Elementary workers not elsewhere classified | 20.2 | 648 |
| | | | |
| 514 | Hairdressers, beauticians and related workers | 14.0 | 3 940 |
| 5141 | Hairdressers | 12.2 | 2 905 |
| 5142 | Beauticians and related workers | 18.8 | 1 035 |
| | | | |
| 832 | Car, van and motorcycle drivers | 13.7 | 9 851 |
| 8322 | Car, taxi and van drivers | 13.7 | 9 752 |

| | | | |
|---|---|---|---|
| 524 | Other sales workers | 12.9 | 19 541 |
| 5242 | Sales demonstrators | 7.8 | 1 687 |
| 5243 | Door to door salespersons | 12.3 | 130 |
| 4244 | Contact centre salespersons | 14.4 | 5 668 |
| 5245 | Service station attendants | 6.8 | 3 003 |
| 5246 | Food service counter attendants | 14.8 | 8 891 |
| | | | |
| 411 | General office clerks | 12.7 | 5 809 |
| – | No further digits applied | | |
| | | | |
| 611 | Market gardeners and crop growers | 12.5 | 5 961 |
| 61112 | Field crop supervisors and workers | 19.7 | 940 |
| 61132 | Gardeners, horticultural and nursery growers and workers | 11.0 | 4 748 |
| | | | |
| 941 | Food preparation assistants | 11.8 | 15 945 |
| 9411 | Fast food preparers | 20.1 | 1 149 |
| 9412 | Kitchen helpers | 11.2 | 14 796 |
| | | | |
| 621 | Forestry and related workers | 11.4 | 2 382 |
| – | No further digits applied | | |
| | | | |
| 235 | Other teaching professionals | 10.9 | 23 569 |
| 2351 | Education methods specialists | 3.2 | 5 760 |
| 2352 | Special needs teachers | 2.3 | 6 677 |
| 2353 | Other language teachers | 21.3 | 221 |
| 2354 | Other music teachers | 17.6 | 1 651 |
| 2355 | Other arts teachers | 26.2 | 854 |
| 23591 | Career counsellors | 3.6 | 2 717 |
| 23592 | Other teaching professionals | 27.9 | 5 606 |
| | | | |
| 622 | Fishery workers, hunters and trappers | 10.3 | 447 |
| 62212 | Fishery supervisors and workers (Aquaculture workers) | 5.9 | 289 |
| 6222 | Inland and coastal waters fishery workers | 15.0 | 127 |
| | | | |
| 612 | Animal producers | 10.0 | 7 079 |
| 61212 | Dairy and livestock workers | 18.1 | 1 677 |
| 61214 | Farm relief workers | 6.7 | 4 460 |
| 6122 | Poultry producers | 10.4 | 173 |
| 61291 | Fur and reindeer producers | 9.7 | 659 |
| | | | |
| 523 | Cashiers and ticket clerks | 9.9 | 7 676 |
| – | No further digits applied | | |

| | | | |
|---|---|---|---|
| 911 | Domestic, hotel and office cleaners and helpers | 9.1 | 63 385 |
| 9111 | Domestic cleaners and helpers | 20.5 | 156 |
| 91121 | Office cleaners, etc. | 9.8 | 37 020 |
| 91122 | Hotel cleaners | 10.5 | 1 405 |
| 91123 | Hospital and institutional helpers | 6.3 | 17 020 |
| 91124 | Kindergarten assistants | 9.1 | 4 211 |
| 91129 | Other cleaners not elsewhere classified | 14.9 | 3 573 |
| | | | |
| 516 | Other personal services workers | 8.7 | 2 205 |
| 51631 | Undertakers and related funeral workers | 11.2 | 250 |
| 5164 | Pet groomers and animal care workers | 11.4 | 964 |
| 5165 | Driving instructors | 4.2 | 739 |
| 5169 | Personal services workers not elsewhere classified | 11.8 | 144 |
| | | | |
| 531 | Child care workers and teachers' aides | 8.4 | 48 839 |
| 53111 | Childminders in kindergartens and other institutions | 4.4 | 24 883 |
| 53112 | Private childminders | 4.4 | 10 242 |
| 53113 | Children's club leaders | 22.9 | 2 003 |
| 5312 | Teachers' aides | 18.2 | 11 480 |
| | | | |
| 512 | Cooks | 7.9 | 32 654 |
| 51201 | Cooks | 10.5 | 20 323 |
| 51202 | Restaurant services supervisors and shift managers | 3.8 | 12 299 |
| | | | |
| 711 | Building frame and related trades workers | 7.9 | 45 736 |
| 7111 | House builders | 9.7 | 21 620 |
| 7112 | Bricklayers and related workers | 10.9 | 1 162 |
| 7113 | Stonemasons, stone cutters, splitters and carvers | 6.5 | 430 |
| 7114 | Concrete placers, concrete finishers and related workers | 4.7 | 1 100 |
| 7115 | Carpenters and joiners | 6.5 | 15 667 |
| 7119 | Building frame and related trades workers not elsewhere classified | 4.1 | 4 619 |
| | | | |
| 522 | Shop salespersons | 7.6 | 94 788 |
| 5221 | Shop keepers | 14.7 | 238 |
| 5222 | Shop supervisors | 2.2 | 10 634 |
| 5223 | Shop sales assistants | 8.2 | 83 916 |
| | | | |
| 912 | Vehicle, window, laundry and other hand cleaning workers | 7.5 | 292 |
| 9122 | Vehicle cleaners | 6.7 | 208 |
| | | | |
| 541 | Protective services workers | 7.3 | 18 881 |
| 5411 | Fire-fighters | 7.1 | 3 682 |
| 5412 | Police officers | 1.3 | 4 200 |
| 5413 | Prison guards | 1.7 | 1 713 |
| 5414 | Security guards | 11.5 | 8 349 |
| 5419 | Protective services workers not elsewhere classified | 8.0 | 937 |

| | | | |
|---|---|---|---|
| 931 | Mining and construction labourers | 7.2 | 9 382 |
| 9311 | Mining and quarrying labourers | 2.6 | 379 |
| 9312 | Civil engineering labourers | 5.4 | 5 979 |
| 9313 | Building construction labourers | 11.6 | 2 789 |
| | | | |
| 511 | Travel attendants, conductors and guides | 7.2 | 2 721 |
| 5111 | Travel attendants and travel stewards | 2.2 | 815 |
| 5112 | Transport conductors | 3.1 | 1 333 |
| 5113 | Travel guides | 23.7 | 573 |
| | | | |
| 264 | Authors, journalists and linguists | 7.1 | 10 826 |
| 2641 | Authors and related writers | 15.3 | 301 |
| 26421 | Managing editors and subeditors | 5.5 | 568 |
| 26422 | Journalists | 4.1 | 4 852 |
| 26423 | Radio and television journalists | 4.8 | 2 473 |
| 2643 | Translators, interpreters and other linguists | 14.3 | 2 622 |
| | | | |
| 753 | Garment and related trades workers | 6.7 | 2 759 |
| 7531 | Tailors, dressmakers, furriers and hatters | 7.0 | 597 |
| 7533 | Sewing, embroidery and related workers | 9.8 | 783 |
| 7534 | Upholsterers and related workers | 4.4 | 956 |
| 7536 | Shoemakers and related workers | 6.4 | 157 |

**Supplementary Table 8.** Predicted proportion of employees with unemployment occurrence and the predicted number of unemployment days during the 5-year follow-up by employment type, stratified by 5-year unemployment history

| | Unemployment history | | | |
|---|---|---|---|---|
| Predicted % unemployment occurrence during 5 years (a) | No unemployment (N=1248285) | 1–90 days (N=184103) | 91–365 days (N=213563) | >365 days (N=151931) |
| Employment type | | | | |
| High income | 9.9 | 26.9 | 39.1 | 46.8 |
| Standard | 11.3 | 29.1 | 39.1 | 49.8 |
| Sub-standard | 16.4 | 37.1 | 49.7 | 63.5 |
| Precarious | 23.9 | 50.1 | 63.8 | 74.5 |
| Limited | 24.5 | 48.6 | 72.6 | 84.6 |
| Predicted unemployment days during 5 years (a, b) | No unemployment (N=155438) | 1–90 days (N=62837) | 91–365 days (N=110068) | >365 days (N=111249) |
| Employment type | | | | |
| High income | 360 | 365 | 427 | 514 |
| Standard | 361 | 326 | 382 | 497 |
| Sub-standard | 396 | 356 | 415 | 583 |
| Precarious | 444 | 407 | 464 | 640 |
| Limited | 456 | 426 | 586 | 807 |

Adjusted for age, gender, origin and education

(a) Including individuals with a defined 3-digit occupation and who remain in the country during the whole 5-year follow-up

(b) Including individuals with unemployment occurrence during the 5-year follow-up
